# tidyGenR: tidy multilocus amplicon genotypes in R

**DOI:** 10.7717/peerj.21726

**Published:** 2026-09-17

**Authors:** Miguel Camacho-Sanchez, Jennifer A. Leonard

**Affiliations:** 1 Conservation and Evolutionary Genetics Group, Estación Biológica de Doñana, Consejo Superior de Investigaciones Científicas, Sevilla, Spain; 2 Institute of Organismic and Molecular Evolution (iomE), Anthropologie (iomE), Johannes-Gutenberg Universität Mainz, Mainz, Germany

**Keywords:** R package, Population genetics, Amplicon sequencing, Genotyping, Tidy data, High-throughput sequencing

## Abstract

Multiplexed amplicon sequencing has become an important tool in phylogenetics and conservation genetics. Amplicon sequencing reads need to be processed to get final haplotypes. The bioinformatics involved is often limiting for embarking on these kind of projects and there are few tools designed to handle this type of data. *tidyGenR* is an R package for reproducible multilocus amplicon genotyping workflows from sequencing reads. It provides a modular workflow that starts by demultiplexing loci, variant determination with *DADA2*, and ends with genotyping. Input data can be raw single-end or paired-end FASTQ reads and the main outputs are haplotypes in tidy tables. Results can also be exported as FASTA files. We successfully tested *tidyGenR* on amplicon libraries of 27 loci from a population genetics study in a rodent. The results from *tidyGenR* were reliable and robust across a wide range of read depths. In addition, *tidyGenR* offers greater flexibility and interoperability.

## Introduction

Sequence-based markers are widely used in evolutionary biology and conservation genetics (Allendorf, Luikart & Aitken, 2012). Multiplexed amplicon sequencing can simultaneously generate data for many loci from many samples (Bybee et al., 2011; Forcina et al., 2021; Stiller et al., 2009). Although reduced-representation sequencing approaches that subsample a fraction of the genome have become increasingly common in conservation genetics (Andrews et al., 2016; Faircloth et al., 2012), multiplexed amplicon sequencing remains preferable in some biological systems to target homologous, non-anonymous markers. This has extended the use of sequence markers to large multilocus phylogenies and phylogeographic studies (*e.g.*, Barrow et al., 2014; Bybee et al., 2011; Hinckley et al., 2020; Lemmon & Lemmon, 2013; O’Neill et al., 2013), to evaluate neutral genetic diversity (*e.g.*, Forcina et al., 2021; Loera-Sánchez, Studer & Kölliker, 2022), and functional variation (*e.g.*, Sommer, Courtiol & Mazzoni, 2013).

A central challenge for the exploitation of amplicon sequencing datasets lies in differentiating real haplotypes from artifacts. The large amounts of data generated by high-throughput sequencing technologies (HTS) along with the inherent errors associated with them (Glenn, 2011) have hindered and slowed the adoption of HTS in conservation genetics. Previous approaches to retrieve variation from amplicon reads included calling SNPs on the amplicons (Barrow et al., 2014), manual genotyping (Camacho-Sanchez et al., 2018) or utilizing specific genotyping software (*e.g.*, *AmpliSAS*, Sebastian et al., 2016; *ACACIA* Gillingham et al., 2021). Genotyping strategies based on read assembly/mapping followed by SNP calling (*e.g.*, GATK workflow), imply complex bioinformatic steps and disregard the nature of duplicated PCR products and errors from amplicon libraries. Specific tools based on discriminating artifacts from real variants have proven effective for getting reliable haplotypes from amplicon data (*e.g.*, Callahan et al., 2016; Sebastian et al., 2016; Scapolatiello et al., 2025). These tools can present practical challenges for end users: user-friendliness, ability to accommodate non-standard input data, modularity, comprehensive parameter tuning, diagnostic tools, code accessibility, and/ or interoperability of data structures. Thus, there is a need for a software tool that combines power with flexibility and ease of use. R (R Core Team, 2024) has become a preferred coding and analytic environment in Evolutionary Biology and Conservation Genetics. In this context, the implementation of amplicon processing tools in R has the extra-benefit of easier adoption by researchers in this field, and enables connectivity with upstream and downstream coding workflows and other R libraries.

We have developed *tidyGenR*, an R package for multilocus genotyping of amplicon sequencing data. *tidyGenR* provides well-documented code with examples, high modularity, and tidy interoperable data structures (Hutchison et al., 2024). The R environment offers a cross-platform solution and easy integration. The package is available on CRAN and at https://github.com/csmiguel/tidyGenR.

## Methods

### Overview

**Figure 1:**
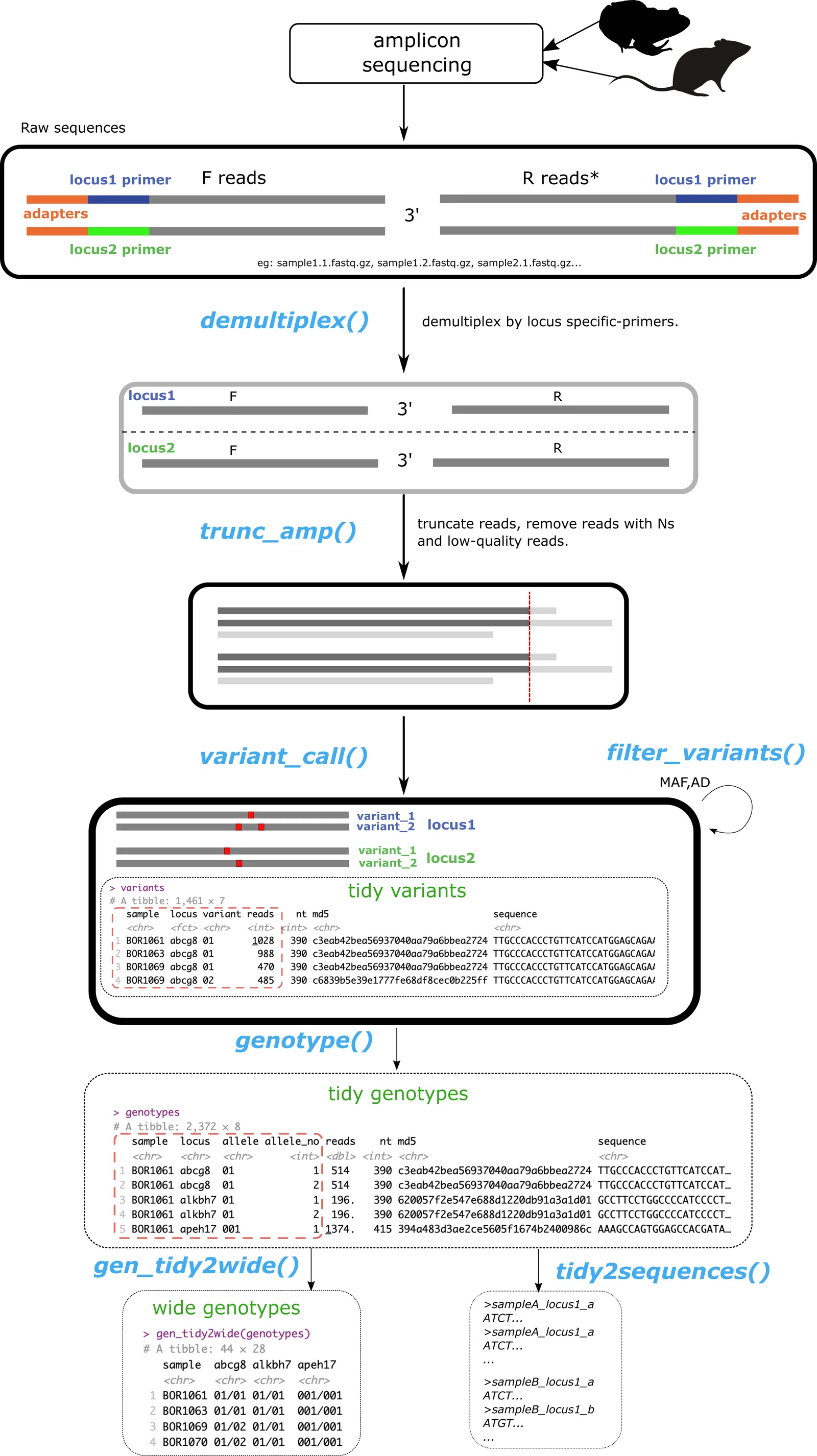
*tidyGenR* workflow. Single or paired-end reads of HTS sequences from multiplexed amplicons are demultiplexed by locus with *demultiplex()*. *trunc_amp()* truncates reads to defined lengths and removes low-quality reads. *variant_call()* determines ASV using *dada2* functions. Variants and genotypes are stored in tidy *data frames* which are easy to filter and reformat. Other functions are described in ‘Methods’. Silhouettes obtained from PhyloPic (https://www.phylopic.org). Both images are released under the CC0 1.0.

*tidyGenR* provides a reproducible and modular framework integrating existing tools for multilocus amplicon genotyping, and accessory functions for diagnosis and reformatting data into interoperable tidy structures (Fig. 1). Variants are called internally using *DADA2* (Callahan et al., 2016). The *DADA2* algorithm was initially designed to retrieve amplicon sequence variants from metabarcoding data. We benefit from its proven performance in denoising amplicon reads (Callahan et al., 2016; and later *DADA2* citations) and extend it to multilocus sequencing data. The package is populated with functions for format conversion, generation of useful outputs and diagnosis. Data have many entry points in the workflow, so some steps can be optionally processed externally. *tidyGenR* can be run on single-end or paired-end data. The variant and genotype output *data frames* meet tidy data principles (Wickham, 2014; Wickham et al., 2019). Compared to ‘*samples x loci*’ genotype matrices used by most genetic software, in *tidy* format each data point is a row. Thus, each record is a variant/allele call for a given sample and locus, and each column is a variable describing characteristics of the variant/allele (Fig. 1).

### Quick start guide

Instructions on how to use the package are provided in the vignettes. Further details on its functionality are provided at https://csmiguel.github.io/tidyGenR_benchmarking/.

Below is a minimal reproducible workflow using single-end Illumina multiplexed amplicon reads to obtain haplotype-based genotypes from three samples:

**Table:** 

“‘
library(tidyGenR)
# raw forward reads
raw <- system.file(”extdata”, ”raw”, package = ”tidyGenR”)
freads <- list.files(raw, pattern = ”1.fastq”, full.names = TRUE)
# demultiplex by locus. cutadapt needs to be on the $PATH
data(”primers”)
dem_dir <- file.path(tempdir(), ”demultiplexed”)
demultiplex(freads = freads, rreads = NULL, mode = ”se”, primers = primers[1:3, ], outdir = dem_dir)
# truncate reads
trunc_dir <- file.path(tempdir(), ”truncated”)
trunc_amp(mode_trun = ”se”, fw_pattern = ”1.fastq.gz”, in_dir = dem_dir, trunc_fr = 100, outdir = trunc_dir)
# variant calling
variants <- variant_call(in_dir = trunc_dir)
# genotype
genotypes <- genotype(variants, ADt = 10, ploidy = 2)
“‘

### Input data

*tidyGenR* processes amplicon sequencing reads from multiple loci simultaneously. It expects Illumina FASTQ files with locus-specific primers at one or both ends. Sample names are extracted from the names of the FASTQ files. So, they need to meet a naming convention: alphanumeric characters followed by a non-alphanumeric character and a specific extension for forward (F) and/or reverse (R) reads (*e.g. sample1.1.fastq.gz* or *Sample01_F.fastq.gz*). The function *tidyGenR::check_raw_reads()* can be used to check: (1) all files have a minimum number of reads, (2) sample names are not duplicated, and (3) there are no orphan F/R files when running in paired-end mode.

### Core steps

#### Demultiplexing

Loci are demultiplexed based on the presence of one or both locus-specific primers at read ends, using *tidyGenR::demultiplex()*, a wrapper function for *cutadapt* (Martin, 2011). Primer sequences need to be provided in a *dataframe.* Demultiplexing can be run on single-end mode, paired-end mode with ‘*paired adapters*’, and with *‘linked primers’* when F/R primers are in one single read. Locus-demultiplexed reads are written to files: ‘*sample.locus.[1*—*2].fastq.gz*’. The function *tidyGenR::remove_poor_fastq()* is recommended after *demultiplex()* to discard files with low counts or no reads.

#### Read truncation

All reads for a given locus must be truncated to the same length and reads with any ambiguous nucleotide discarded before variant calling. Already-merged sequences from paired-end sequencing do not need to be truncated. Truncation lengths can be set using previous knowledge from per-base quality plots to maximize the amount of information retained. The function *tidyGenR::trunc_amp()*, a wrapper function of *dada2::trimAndFilter()*, can be used to truncate reads from each locus. It extracts sample and locus names from the demultiplexed FASTQ files and applies global or specific truncation lengths per locus. It writes truncated reads and returns a list of matrices with the number of reads that passed the filters. Reads containing *N*s or below a read quality or length threshold are discarded*.*

#### Variant calling

Variant calling will determine Amplicon Sequence Variants (ASV). Thus, it will try to detect variants differing down to 1 nt (Callahan et al., 2016). ASVs are called using the wrapper function *tidyGenR::variant_call()*. For each locus, it runs *dada2 learnErrors()*, *dada()*, *mergePairs()* (for paired-end data), *removeBimeraDenovo()*, *makeSequenceTable()*, and performs reformatting and filtering of variants. The sensitive parameters “OMEGA_A” and “BAND_SIZE” will work well with default values in most cases, but they can be diagnosed with *explore_dada()* and be modified inside *variant_call()* (see optimization section below). Per-base errors are modelled and used to denoise sequences with *DADA2*, independently for F and R reads within each locus. Bimeras are removed and variants are reformatted to a tidy *dataframe*. A function has been implemented to model errors from binned Illumina Q-scores, as those from NovaSeq 6000, NextSeq 1000, NextSeq 2000, and iSeq. This function can be called with *loess_err_mod4* and called natively in *dada2* as of commit #7714487, December 24 (https://github.com/benjjneb/dada2/issues/1307). Non-overlapping F/R reads can be concatenated with ten ambiguous nucleotides (*N*s) by activating *c_unmerged*. This workaround can yield more variants when F/R reads do not overlap but it will generate artificial haplotype sequences, so it needs to be used carefully. Variants can be filtered by applying per-sample Minimum Allele (variant) Frequency (*MAF*) and Allele (variant) Depth (*AD*). These filtering options are added as attributes to the output *dataframe*. Variants are stored in a *tidy* table with seven variables: *sample* (sample name), *locus* (locus name), *variant* (variant name), *reads* (number of reads supporting a given variant for given sample), *nt* (length of the haplotype), *md5* (MD5 hash of the DNA sequence), and *sequence* (DNA sequence). The MD5 hash is a fixed-length 128-bit identifier generated from the DNA sequence. Because identical sequences produce identical hashes, MD5 identifiers allow unambiguous tracking of sequence variants throughout the workflow independently of sequence length. They also facilitate efficient linking and comparison of intermediate tables across the workflow.

#### Genotyping

The role of *genotype()* is to reformat sequence variants into alleles given a “ploidy” expectation. The *ploidy* argument can be set to numeric *“1”, “2”* or “*poly”*, which determines the maximum number of expected alleles per locus. When ‘*ploidy*=* 2’* the Allele Depth Threshold (*ADt*) is used to discriminate hemizygotes (only one allele is known) from homozygotes. Reads (‘reads’) supporting each variant are divided equally between alleles in homozygosis. The output is a tidy table with at least four columns: *sample*, *locus*, *allele*, *allele_no*. The column *variant* is replaced with *allele*, and *allele_no* contains information on the allele ID for each locus. The attribute *ploidy* is added to the output table. If *ploidy*=* “poly”*, no default values of ploidy are assumed.

### Evaluation and optimization of variant calls

Two diagnosic functions have been included. Default *DADA2* sensitive parameters are effective in determining real variants (Callahan et al., 2016). However, the prior knowledge of the maximum number of alleles expected for each locus, *e.g.*, two in case of diploid autosomal markers, can be used for fine tuning of variant calling. These can be explored with *tidyGenR::explore_dada()*, and different calling strategies can be compared with *tidyGenR::compare_calls*. The function *explore_dada()* performs *DADA2* runs under a given set of parameters and outputs the relevant statistics and diagnosis plots. It computes the *log(-log(birth_pval))* for each variant, which is proposed in Rosen et al. (2012) to visualize *birth_pval*. This parameter is the probability that the error model gives to an observed ‘child’ variant being the same as its parent sequence. If *birth_pval* is lower than the *OMEGA_A* threshold (see *variant_call()*), the child variant is promoted to its own cluster. The plots produced by *explore_dada()* represent variant frequencies against their *log(-log(birth_pval))*. This representation can guide users in making decisions on *OMEGA_A* and variant frequency filters (see vignettes). The function *compare_calls()* compares results from any number of tidy variants or genotype objects with shared samples and loci. It helps to visualize and compare which variants were/were not called with different set of parameters by returning heatmaps and tables.

The variants can be aligned against a reference list of FASTA sequences to assess their homology. We have implemented this functionality in *align_variants_ref()*.

### Genotype re-formatting

A family of functions starting with “*gen_*” reformat the genotype data between three types of *data frames*: (1) *tidy*, one row per allele/locus/sample; (2) *compact*, one row per locus/sample; (3) *wide*, rows are samples and columns are loci and each cell contains a genotype call. Genotypes can also be exported to a *STRUCTURE* (Pritchard, Stephens & Donnelly, 2000) ‘str’ text file with *gen_wide2strucutre()*, and to a file readable by *GENALEX* (Peakall & Smouse, 2012) with *gen_tidy2genalex()*. Genotypes from the program *AmpliSAT* can be imported with *amplisas2tidy()*.

### Other functions

The package is complemented with other functions for manipulation of variants/ genotypes, and generation of outputs (Table 1).

**Table 1:** Additional functions included in *tidyGenR*.

Manipulation of variants and genotypes	
*remove_hemizygotes()*	Remove hemizygous calls from tidy genotypes.
*remove_monomorphic()*	Remove monomorphic loci from tidy genotypes.
*rename_alleles()*	Rename alleles in tidy genotypes based on reference names.
Generate outputs	
*tidy2sequences()*	Exports tidy variants or genotype sequences to a FASTA file or to a *DNAStringSet.*
*dereplicate()*	Computes frequency of dereplicated DNA sequences. (*e.g.*, File S1).
*out_popart()*	Exports multiple sequence alignments to NEXUS readable by PopART (https://popart.maths.otago.ac.nz/).
*reads_loci_samples()*	Outputs *dataframe* with number of reads per sample (rows) for each locus (columns) from tidy variants or FASTQ files (*e.g.*, Tables S2 and S3)
*reads_track()*	Track the number of reads across the pipeline for each samples (*e.g.*, Table S1).

### Data

*tidyGenR* is distributed with data from a population genetics study of the summit rat, *Rattus baluensis* (Camacho-Sanchez et al., 2026). This includes: ‘*primers’*, table with 27 primer pairs to demultiplex raw FASTQ files; ‘*trunc_fr’*, table with optimal truncation lengths for forward and reverse reads; *variants*, filtered tidy variants from 27 loci for 44 individuals of *Rattus baluensis* from a single population; ‘*genotypes’*, tidy genotypes for those same; “extdata/metadata.csv”, sample metadata; “extdata/reference_alleles.fasta”, name of reference alleles. A tiny dataset from FASTQ files of three loci from three random samples has been included in “extdata” for testing and examples.

### Dependencies

*tidyGenR* depends on the native pipe from R ≥ 4.1.0, *DADA2* ≥ 1.16 (Callahan et al., 2016) for denoising and core functions, *ShortRead* (Morgan et al., 2009) and *Biostrings* (Pagès et al., 2024) for manipulation of DNA sequences, *DECIPHER* (Wright, 2016) for sequence alignment in R, *digest* (Eddelbuettel, 2024) for generating DNA sequence hashes, *plyr* (Wickham, 2011) and multiple packages from the *tidyverse* (Wickham et al., 2019) for data manipulation: *dplyr, stringr*, *readr*, *tibble, tidyr*, *tidyselect*. Plotting is carried out with *ggplot2* and *patchwork* (Pedersen, 2024) and writing output to EXCEL with *writexl* (Ooms, 2025). *Cutadapt* is called for demultiplexing by locus, and only versions ≥ 2.0 implement demultiplexing in paired-end mode and with linked-adapters (beta mode). If *cutadapt* is not installed, *demultiplex(run*=* TRUE)* will fail, but the error can be bypassed, and a bash script be produced if *run* is turned off (run = FALSE).

### Benchmarking: genotyping a panel of introns in a rodent population

The performance of *tidyGenR* was evaluated with amplicon sequences from a rodent population. We evaluated several aspects of *tidyGenR*: (1) its capacity of producing a fully reproducible end-to-end workflow, from raw FASTQ files to sequence-based genotypes, (2) the effect of tunable parameters on variants called, (3) the effect of coverage, (4) the biological plausibility of genotypes, and (5) its performance against other software with similar capabilities. Code and further details for all the steps can be found in https://github.com/csmiguel/tidyGenR_benchmarking.

#### Benchmarking dataset

*tidyGenR* was used to genotype 44 individuals of the rodent *Rattus baluensis* from an isolated population on Mount Trusmadi, northern Borneo, Malaysia. The input reads corresponded to 300PE MiSeq Illumina amplicon sequencing reads from 27 introns multiplexed per library. These samples were part of a population genetics study published in Camacho-Sanchez et al. (2026). Amplicon libraries were built in two-steps: 1° , amplification of introns in a single multiplex PCR with adjusted primer concentrations; 2° , adapter ligation by PCR. Sequence lengths of these markers in *R. baluensis* range from 370 nt (*ms4a2*-5) to 486 nt (*nkfbia*-5) (Camacho-Sanchez et al., 2018). Libraries were sequenced at John Hopkins University. Sequencing reads were demultiplexed by sample individual barcodes.

*Pre-processing*. Sample raw FASTQ files (NCBI project PRJNA680166) were renamed to comply with file naming for *tidyGenR*. Adequacy of input FASTQ was checked with *check_raw_reads()*. Primers are stored in *data(“primers”).* These primers were supplied to *demultiplex()* to demultiplex by locus in paired-end mode invoking *cutadapt* 4.1. Demultiplexed FASTQ files less than 10 reads were removed. Quality of demultiplexed reads was explored with FastQC v0.12.1 (http://www.bioinformatics.babraham.ac.uk/projects/fastqc) and the reports aggregated with MultiQC v1.25.1 (Ewels et al., 2016). Given the rapid drop in quality in R reads (Fig. S1), we set asymmetric truncation lengths of 270 nt and 180 nt for F and R reads, respectively. Reads were truncated in paired-end mode and minimally filtered with *trunc_amp(max_ee*=* c(4, 5)*, *trunc_q*=* 2)*.

#### Optimization of variant calling

**Figure 2:**
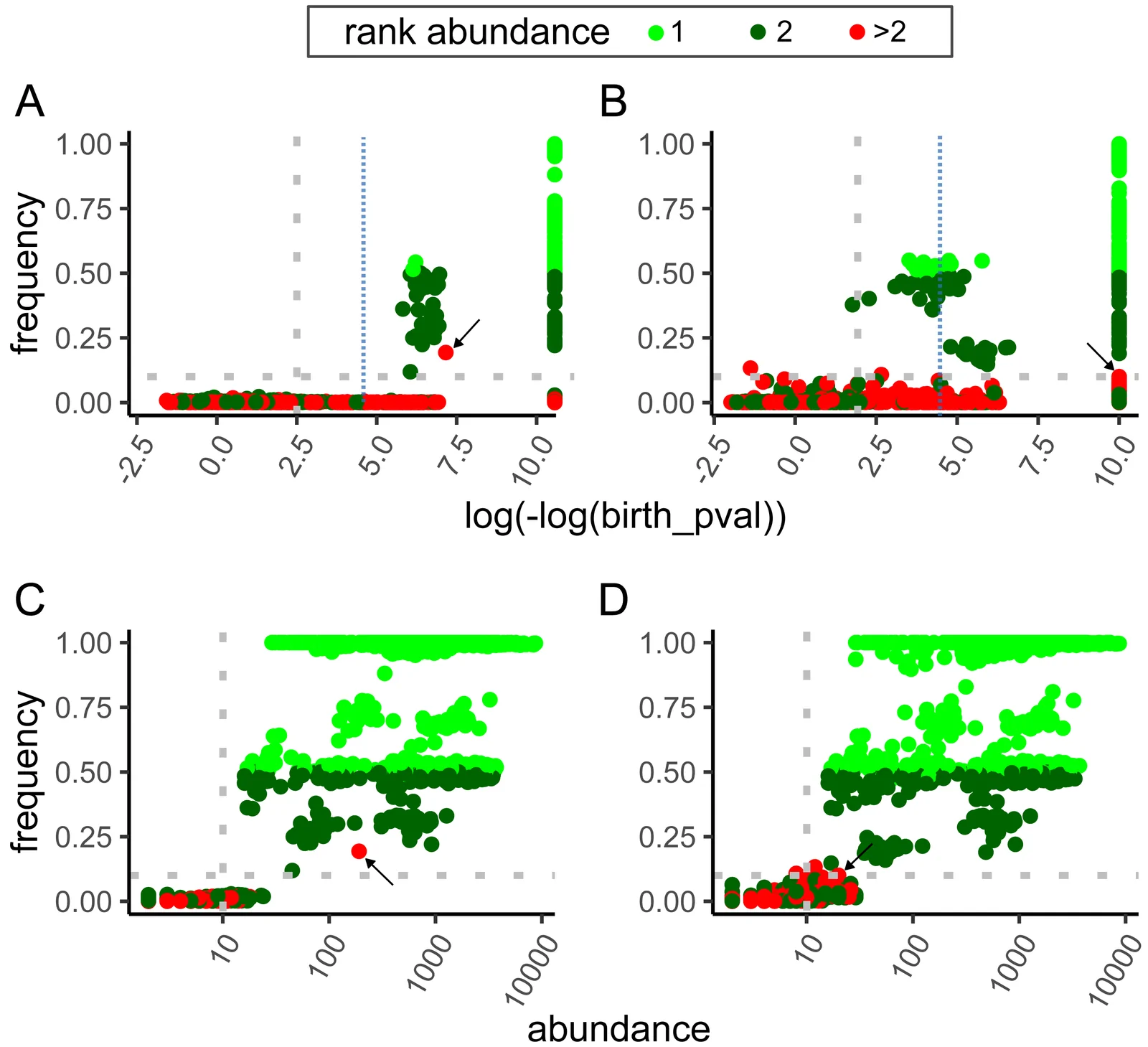
Variant calling diagnosis. Exploration of *DADA2* variant clustering for forward (A, C) and reverse (B, D) reads. Each point represents a sequence variant in a locus and sample. The top-right quadrants in each plot show the variants considered to be real under putative user-defined thresholds (dashed grey lines; being the default value the blue dashed line). The *y*-axis shows variant relative frequencies. The *x*-axis shows total abundance (C, D) or the strength of evidence against the variant being explained as a sequencing error, expressed as *log(–log(birth_pval))* following the *DADA2* error model. Variants are colored by abundance rank within each locus and sample. Arrows highlight variants with atypical behavior that resulted in downstream genotype loss.

*OMEGA_A* sets the threshold for *DADA2* to call “child” variants. Fine tuning of *OMEGA_A* was explored with *explore_dada()*. After checking *birth_pval* distribution (Fig. 2; Figs. S2–S5), *OMEGA_A* was increased from default value to 0.01 [*log(-log(0.01)) ∼1.52* ] to allow a few more variants to be called at little risk of calling artifacts (Fig. 2). Other parameters that could affect variant calling are *BAND_SIZE* and *pooling*. *BAND* _SIZE restricts the alignment to a band of a given size around the main diagonal of the alignment matrix when Needleman-Wunsch alignments are performed (Needleman & Wunsch, 1970; Rosen et al., 2012). ‘*BAND_SIZE*=* 0*’ disables banding, triggering a full dynamic programming alignment. That is, *BAND_SIZE*=* 0* would consider indels at the ends of the sequences. *Pooled* variant call performs a joint denoising and variant calling for all samples, potentially increasing the power of detecting low-frequency variants (Callahan et al., 2016; https://benjjneb.github.io/dada2/pool.html).

Four runs of *variant_call()* were performed combining ‘*pooling/ non-pooling*’ samples and *BAND_SIZE* of 16 (default) and 0. Variants with a frequency below 0.1 were filtered out, minimizing false positives while retaining most true variants. A threshold for minimum read count was set to 10. Diploid genotypes were forced with *genotype(ploidy*=* 2, ADt*=* 10)*. The runs were compared with *tidyGenR::compare_calls()*. Mismatching variants and genotype calls between methods were investigated manually to detect potential sources of error associated with each strategy. This involved inspecting the multiple sequence alignments of the ASVs and contrasting their abundance against the original frequencies of the dereplicated raw sequences (File S1).

Differences in locus coverage and allele bias were assessed with models using *lme4* (Bates, Maechler & Bolker, 2012) in R. A full model was built in which locus and variants nested in loci were included as fixed effects and sample as a random effect: *glmer(reads ∼locus/variant + (1 — sample), data*= *variants, family*= *“Poisson* ”*).* The fitted model was compared with a null model without the fixed effects. The predicted read coverage for all cases in the input data were plotted.

#### End-to-end workflow

We evaluated the ability of *tidyGenR* to provide a reproducible end-to-end workflow from raw FASTQ reads to final sequence-based haplotypes using Quarto (Allaire et al., 2025) documents rendered at https://csmiguel.github.io/tidyGenR_benchmarking/.

#### Effect of coverage

The capacity of *tidyGenR* to call haplotypes under a range of sequencing depths was evaluated under simulated coverages of 500x, 250x, 125x, 70x, 50x, 35x, and 20x. First, we selected the 20 loci which had an average coverage per sample above 500x. Their FASTQ files were subsampled with *ShortRead* samplers to the target number of reads. Variants were called over these subsets. Final genotypes between subsets were compared with *compare_calls()* and with four metrics: (1) *observed heterozygosity* (*Ho*), since lower coverage could hinder the determination of the less frequent allele, leading to an increase in homozygosity, (2) *missingness*, measured as missing genotype calls (*i.e.,* cells in the ‘*sample x locus’* matrices with no data), (3) *allele similarity*, quantified as the proportion of alleles that are identical to the subset with more alleles genotyped, and (4) *distinct haplotypes*, or total number of distinct alleles considering all loci.

#### Comparison of tidyGenR and AmpliSAT

The results of *tidyGenR* were compared against those from *AmpliSAS*, a tool with shared capabilities and which has been the most popular option with amplicon datasets (115 CrossRef citations by July 2025)*.* In detail, artificial 6-nt barcodes were added to the raw sequences in R using *DNABarcodes* (Buschmann, 2017). FASTQ files from all samples were concatenated into unique F and R files. The metadata file required for *AmpliSAT* (*locus name, forward and reverse primers, amplicon length, barcodes*) was created with custom R scripts. *AmpliSAT* modules were run in a *docker* container (*sixthresearcher/amplisat*) using *podman* 4.9.1 (https://podman.io/). Paired reads were merged with *ampliMERGE* and cleaned with *AmpliCLEAN* using minimum PHRED read quality of 17 and minimum length of 200 nt. Loci metadata (*-d amplicon-data.csv*) were further used to detect non-target amplicons. *AmpliSAS* was run mirroring parameter used in *tidyGenR, i.e., “min_amplicon_seq_depth,all,10”* and *“min_amplicon_seq_frequency,all,10”* (results in File S2). *AmpliSAT* results were read into ‘tidy variants’ with *amplisas2tidy()*, and genotyped as above. The genotypes from *AmpliSAS* were compared with those from tidyGenR using *compare_calls*.

#### Reliability of the haplotype-based genotypes

Genotype likelihoods analogue to those produced by SNP callers are not readily applicable to amplicon data, since most amplicon artifacts are produced at PCR. However, such callers model probabilities for genotypes from sequencing derived stochasticity. Therefore, we took alternative approaches to evaluate the biological plausibility of genotypes. The recurrence of the same allele across different samples is a good indicator of reliability (Sommer, Courtiol & Mazzoni, 2013). Therefore, for each allele we computed the number of presences across samples. Secondly, we checked for the concordance between previously published haplotypes for the same species (Camacho-Sanchez et al., 2018). Lastly, we checked for departures from Hardy-Weinberg equilibrium (HWE). The data analysed comes from a single genetic population (Camacho-Sanchez et al., 2026). Locus-wise deviations from HWE could reflect issues in the genotyping workflow. For each locus, we evaluated departures of observed (*Ho*) and expected heterozygosity (*He*) through *Fis* (Nei, 1987), using *hierfstat* (Goudet, 2005), and departures from HWE with *χ*^2^tests on the observed and expected genotype frequencies, using *genetics* (Warnes et al., 2021).

## Results

### End-to-end solution: genotyping from amplicon sequencing reads

We used tidyGenR to get allele sequences and genotypes from 27 multiplexed loci sequenced in a MiSeq 300PE Illumina run, from 44 rodent samples. Average input reads per sample ranged from 51,500 to 195,000 (mean = 88,300; *s.d.* = 28,900) (Table S1). After demultiplexing with locus-specific primers, average reads per sample decreased to 75,500 (*s.d.* = 23,500). Reads that passed minimum truncation length and minimal filtering (no Ns and minimal quality), decreased the dataset down to an average of 45,200 reads (*s.d.* = 14,200) per sample. Variant calling and variant filtering removed around a further 1% of the reads yielding an average of 44,800 reads/ sample (*s.d.* = 14,100). Diploid genotypes were successfully called for all loci and samples, except for two calls (2/1088 = 0.17% missing calls): *tmem87a* in BOR1091 and *abcg8* in BOR1043 (Fig. S6). These corresponded to cases in which three variants passed all filters. Nevertheless, a maximum of two alleles is expected for these diploid markers, so *tidyGenR::genotype(ploidy*=*2)* forced the introduction of missing values to prevent populating the genotypes with false positives.

The average number of reads supporting each allele was 831 (*s.d.* = 719). Coverage between loci was highly variable. So, variants for some of loci were with less than 100 reads (*i.e.,* in *chrna9*, *nfkbia* and *tmem87a*), while others had over 5,000 reads (*i.e., dhrs3*, *klc2*, and *p2rx1*) (Fig. S8). However, allele balance (similar coverage for both alleles in heterozygous calls) was homogeneous in most cases (Table S3). This was reflected by the mixed models, which improved (ΔAIC 1.3e6) after including the fixed effects locus and variant (*χ*^2^ = 1.3e6, *df* = 48, *p* < 0.001) (Fig. S8).

These *tidyGenR*-produced genotypes permitted the characterization of the population structure and diversity of *Rattus baluensis* on Mount Trusmadi (Camacho-Sanchez et al., 2026).

### Parameter exploration/sensitivity

Default parameters ‘(*maf*=* 0.1, ad*=* 10, OMEGA_A*=* 1e−40; BAND_SIZE*=* 16*)’ recovered most variants with little risk of calling artifacts (Fig. 2). However, relaxing the threshold at which new clusters are formed (*OMEGA_A*) to 0.01 slightly increased the number of variants recovered with little risk of calling artifacts (Fig. 2; Figs. S2–S5; File S3). When disabling ends-free alignment (*BAND_SIZE*=* 0*), new variants with indels at the 5′ and 3′ ends in loci *p2rx1* and *rgd735029* were detected, although this was a specific case from our dataset (Fig. S7). ‘Pooled’ variant calling did not affect results. For our dataset, a minimum frequency of 0.1 retained ‘real’ variants with little risk of calling artifacts.

### Effect of coverage on variants called

The down-sampled FASTQ files at lower coverages revealed genotype calls were identical from 500x down to 50x. At 20x, a few artifacts were introduced: two distinct haplotypes were called and heterozygosity decreased from 0.156 to 0.150. This caused a total of six allelic differences out of 1,015 allele calls evaluated (Table S4; Figs. S9–S10).

### Biological plausibility of genotypes

Most haplotypes, 46 out of 48, determined with *tidyGenR* were shared by two or more individuals. From these, 32 had previously been described in other populations, while 16 were new for the species. Observed and expected heterozygosities were very similar (*Fis* close to zero) for all loci, and *χ*^2^ tests revealed none of them deviated from HWE (Table S5). Only *fetub* deviated from these results, which could be related to co-amplified multiple loci (see also Camacho-Sanchez et al., 2018).

### Comparison of *tidyGenR* with *AmpliSAT*

**Figure 3:**
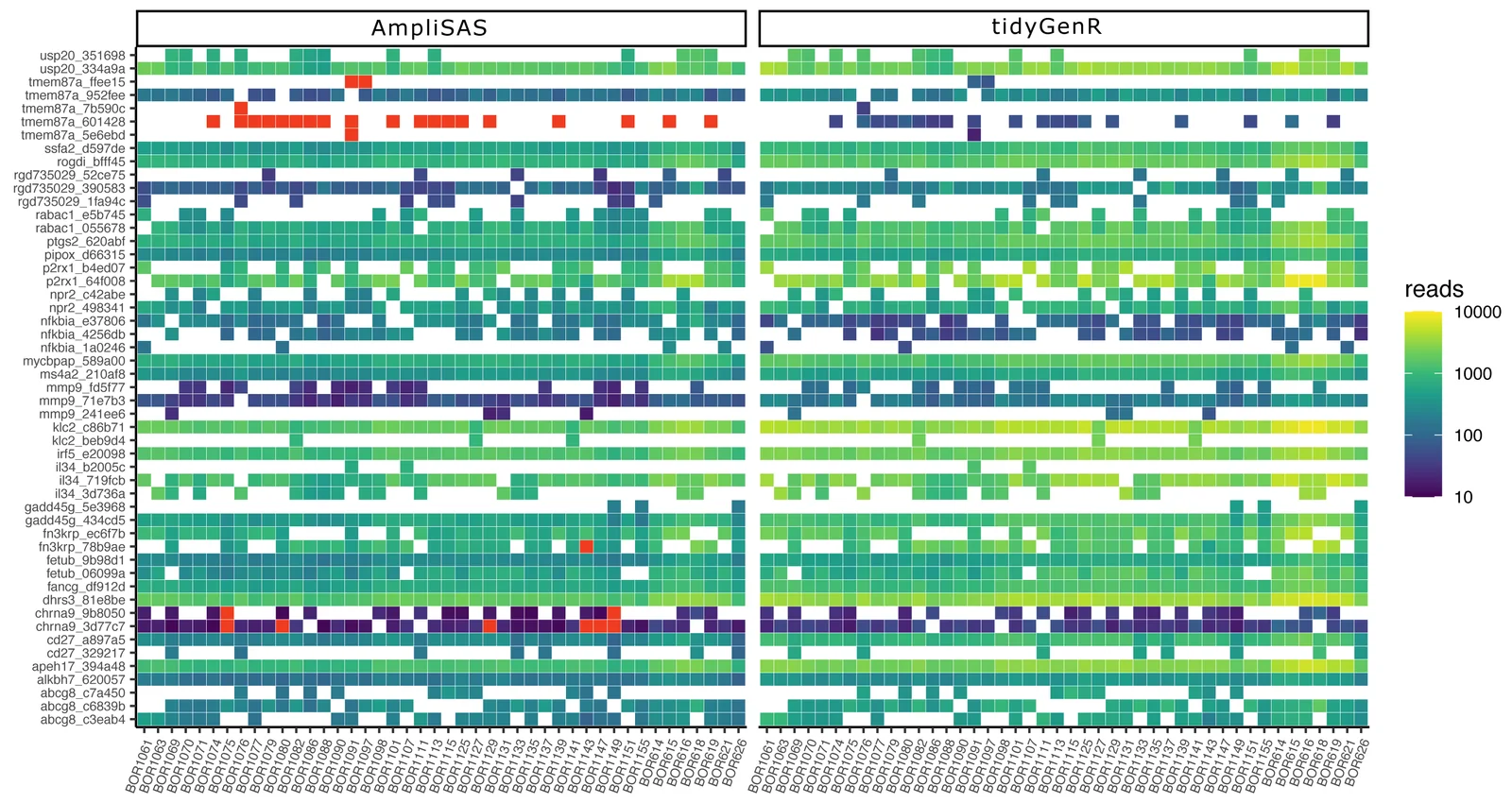
Comparison of haplotypes called with *AmpliSAT* (left) and *tidyGenR* (right). Optimal parameters for the tidyGenR workflow were used (no pooling, band size = 0, Ω *a* = 0.01). Read abundance is color-coded in a log scale gradient. Variants are listed on the *Y*-axis coded as locus name followed by the first six characters of the MD5 hash of its DNA sequence. Red tiles indicate missing calls as compared to the other strategy.

*tidyGenR* and *AmpliSAT* genotypes only differed in 29 out of the 2,376 (1.3%) allele calls. The differences were concentrated in loci with low coverage (*tmem87a*, *chrna9*) where *AmpliSAT* did not call alleles under these parameters (Fig. 3). The number of reads supporting variants in each locus was on average 2.2 (*sd* = 0.69) times larger in *tidyGenR* calls as compared to *AmpliSAT* (Fig. S11).

## Discussion

We developed *tidyGenR*, an R package for reproducible multilocus amplicon genotyping workflows within the R ecosystem. *tidyGenR* integrates demultiplexing, sequence variant inference, filtering, and ploidy-aware genotype formatting into a modular workflow designed for wildlife genetics. By returning outputs in tidy data formats, the package facilitates interoperability with downstream tools commonly used in ecological and evolutionary analyses.

### Innovation and contribution

*tidyGenR* features some advantageous characteristics over its predecessors: (1) the R environment facilitates the connection of the workflow with other R tools; (2) a highly modular workflow with functions that allow fine-tuning of the variant calling and multiple entry points for the data, enabling users to process data with external tools and reintegrate it into the pipeline (Fig. 1); (3) results for variants and genotypes are reported in tidy tables (Wickham, 2014), which are easy to manipulate, highly interoperable, and flexible to scale-up column-wise and row-wise without altering their core structure; (4) the package is complemented with functions to assess its performance, genotype format conversion, variant/ genotype manipulation and output generation of tables and figures; and (5) all functions are well documented with working examples and sample data.

Thus, *tidyGenR* adds to the growing body of R packages with the *tidyomics* philosophy that turn bioinformatics in R into a more accessible and interoperable ecosystem (Carter et al., 2025; Hutchison et al., 2024).

### Evaluation of the methodological approach

Locus demultiplexing relies on *cutadapt* 2.0. We implemented it for its great flexibility to handle primer sequences at 5′ and 3′ ends, and its multiple demultiplexing modes when F/R primers are present. Alternative tools for demultiplexing with lower efficacy and flexibility are *SeqKit2 amplicon* (Shen, Sipos & Zhao, 2024), “separate sequences by barcode” in Geneious (Kearse et al., 2012) and others (Wilkins et al., 2021). The core algorithm for the determination of variants from amplicon sequences is *DADA2*. This denoising algorithm clusters sequences into AVSs: the initial cluster with all sequences is partitioned iteratively into new clusters, stopping when the distance of each sequence to the center of its cluster can be explained with the error model and user-defined *p*-thresholds (Callahan et al., 2016; Rosen et al., 2012). It is the first time to our knowledge that ASVs are proposed and used successfully to assess genetic variants from multilocus diploid datasets, and it is likely the best bioinformatics approach to discriminate real variants from artifacts in amplicon data. There are alternative amplicon denoising algorithms which are not implemented in R such as *AmpliCI* (Peng & Dorman, 2020), a good alternative worth exploring and possibly superior to *DADA2* because of the way it manages individual sequence quality. OTU-like clustering algorithms (*e.g.*, UPARSE-OTU) aim to find representative sequences from clusters, and are therefore not appropriate for finding true sequence variants (Callahan, McMurdie & Holmes, 2017). We only included a direct genotyping in which variants are determined as alleles based on user-input expected ploidy (only 1, 2 or “poly”, are allowed). More complex genotyping models, such as the decision tree proposed by Sommer, Courtiol & Mazzoni (2013) and already present in *AmpliSAT* could be powerful when sample replicates are available. The R environment facilitates custom genotyping decision trees.

#### Benchmarking and optimization

*tidyGenR* was benchmarked by genotyping 27 single-copy loci (except *fetub*) in 44 diploid individuals of the summit rat *Rattus baluensis* from amplicon libraries sequenced on an Illumina MiSEQ 300PE. *tidyGenR* variant calling was robust to large variance in locus coverage. This large variance is common in amplicon libraries from multiplexed PCRs (Forcina et al., 2021). Five loci had variants with indels; three of them homopolymers (*tmem87*, *npr2* and *rgd735029*) and two with simple indels (*p2rx1* and *mmp9*). We did not observe a lower probability of detecting indel-based variants compared to those based on SNPs alone (SF2 and 4). However, fewer reads, as observed in *chrna9*, *tmem87a* and *nfkbia*, were associated with lower probabilities of being identified as new clusters (Figs. S2–S5) below a given threshold (see below).

Diagnosics with *explore_dada()* were helpful to set optimal *OMEGA* _*A* and frequency thresholds for final *variant_call()*. Indeed, *OMEGA_A* was changed from the default 10^−40^, commonly used in metabarcoding (Rosen et al., 2012), to 10^−2^, leading to a gain of a few true positives and no compromise in accuracy (Fig. 2). Lower quality reverse reads increased the coverage of artifacts and lowered the probabilities of true variants being detected (Fig. 2). Therefore, in the case of very noisy data, we recommend assessing the trade-off between trimming low-quality ends and keeping informative sites. The implementation of locus-specific truncation lengths in *trunc_amp()* allows maximization of sequence-recovery by setting locus-specific trimming. Non-overlapping paired-end reads can be artificially merged by turning on *c_unmerged* in *variant_call()*, but this setting needs to be used cautiously as it creates artificial haplotypes*.* Indels were found at the end of haplotypes from locus *p2rx1*. The variation was lost when end-free alignment was run (*i.e., BAND_SIZE* >0), and only recovered when banding was turned off (Fig. S7). The omission of variants caused by the end-free alignment (the default *DADA2* behavior) is designed to discard variation due to small variation in sequence lengths in an alignment. Thus, flanking gaps of a given size >0 set by the *BAND SIZE* parameter have no penalty. This is useful in cases where similar original variants with varying lengths can occur; for instance, derived from variable 3′-end quality trimming or from fixed truncation lengths when there are internal indels. However, when the confidence on the flanking regions from the variants is high (*e.g.*, overlapping paired-end sequences), disabling free-end alignment will allow to account for these flanking gaps in the alignment as real variation. That setting can help to identify variants with indels in loci where indels are expected as with introns. Turning pooling off yielded the same variants, which confirms *tidyGenR* would produce reliable haplotypes when working with samples from different species.

Our *genotype()* function just sets strict rules for reformatting variants into alleles and genotypes given an expected ploidy. The nature of the PCR reactions behind the library preparation hinders the incorporation of statistical models to compute genotype probabilities (see also *Limitations* below). However, the generation of tidy data structures facilitates the integration of custom post-hoc steps to further validate the genotypes. We did by assessing the biological plausibility of the genotypes in R.

### Reliability: effect of coverage and biological plausibility of genotypes

Coverages from 50x and above produced robust results: identical genotypes across sub-sampled sets (Table S4; Figs. S9–S10). At 20x, we recorded very few cases of different genotype calls, associated with either new haplotypes being introduced (*n* =2) or loss of heterozygosity (*Ho* from 0.156 to 0.150). The loss of heterozygosity was expected, since at low coverage it is less likely to sample both alleles given the underlying binomial distribution. The two new haplotypes introduced at 20x were likely overrepresented artifacts derived from sampling random sequences.

The credibility of the haplotypes was determined to be high after comparing shared alleles between these and previously published genotypes. Thus, 46 out of the 48 alleles were shared by two or more individuals, and from those 32 had already been published in other populations of the same species (Camacho-Sanchez et al., 2018). In addition, genotype frequencies across loci adhered to expectations under HWE (Table S5).

### Comparison with AmpliSAT

*tidyGenR* results were very similar to *AmpliSAT* under the parameters used. We detected some differences. First, *tidyGenR* genotype calls were more complete as it was able to call variants in the loci with lowest coverage: 29 extra cells in the variant matrix (*n* = 2,376 cells, ‘*sample x variants’*) (Fig. 3). Secondly, the number of reads supporting each variant was on average 2.2 times larger in *tidyGenR* (Fig. S11). Since the denoising algorithm clusters sequences which can differ up to a distance set by the error model, many of the variants discarded by *AmpliSAT* as artifacts are added to real variant clusters while denoising with *DADA2* (Callahan et al., 2016). We tried to mimic the *AmpliSAT* run with analogue parameters in *tidyGenR*, but we did not explore a wider parameter space in *AmpliSAT*. Possibly a fine-tuning of the clustering and filtering parameters in *AmpliSAT* could lead to results like those we describe for *tidyGenR*.

### Limitations and future directions

Errors are assumed to be independent inside reads and between reads (Rosen et al., 2012). Early PCR errors violate this assumption, potentially leading to overrepresented artifacts being detected as true variants. This could be the case in our dataset for the third variants that passed filters in *abcg8* in BOR1143 and *tmem87a* in BOR1091 (Fig. 2). Replicated libraries from independent PCRs (Salado et al., 2021; Sommer, Courtiol & Mazzoni, 2013) or including non-degenerate multiple primer pairs per locus (Marmesat et al., 2016) are effective strategies to control for errors associated with PCR duplicates, to detect variants in loci with strong allele bias, and to control for allelic dropout. The tidy data structure of variants and genotypes permits easy inclusion of alternative genotyping methods, such as in Sommer, Courtiol & Mazzoni (2013), which would make *tidyGenR* a good alternative for detecting alleles in multi-copy genes. *tidyGenR* could potentially be used with long-read amplicon sequences from Nanopore or PacBio, since sequence quality from these technologies has been increasing in the last years (Karst et al., 2021). However, the way *DADA2* would model errors in these potentially high-error prone reads needs to be evaluated. Alternatively, other denoising algorithms such as *AmpliCI* could be run outside *tidyGenR*. The core denoising algorithm *DADA2* is not designed to model errors from microsatellite repeats. There is a variety of well performing tools such as MEGASAT (Zhan et al., 2017) and SatAnalyzer (Liu et al., 2024), that utilize prior knowledge of flanking regions, identity of the sequence repeat, and frequency distribution of sequence lengths to semi-automatize genotyping of this type of sequences. This straightforward approach, where no error modelling is applied, could be implemented in future versions of *tidyGenR* to take advantage of downstream tidy data structures.

## Conclusions

The price of HTS has decreased substantially and is now accessible to very many projects. *tidyGenR* provides a reliable tool for reproducible multilocus amplicon genotyping workflows in the R ecosystem. It comes with reproducible examples and well-documented functions with the aim of making HTS amplicon analysis accessible to a wider group of evolutionary and conservation geneticists.

##  Supplemental Information

10.7717/peerj.21726/supp-1 Supplemental Information 1 Supplemental Tables ST1. Reads across the pipeline; ST2. Number of demultiplexed reads per locus and sample; ST3. Number of reads supporting filtered variants for each locus; ST4. Effect of coverage; ST5. Heterozygosity and HWE.

10.7717/peerj.21726/supp-2 Supplemental Information 2 Supplemental Figures SF1 read quality; SF2-5, exploration of *DADA2* clustering per locus; SF6. Comparison of genotypes *versus* variants for the best strategy; SF7. comparison of calling strategies; SF8. Fitted reads supporting variants; SF9. Effect of coverage, comparison across subsets; SF10. Effect of coverage, summary statistics; SF11. Reads supporting variants *AmpliSAT* vs *tidyGenR*.

10.7717/peerj.21726/supp-3 Supplemental Information 3 Dereplicated sequences from demultiplexed truncated filtered FASTQ

10.7717/peerj.21726/supp-4 Supplemental Information 4 * AmpliSAT* results

10.7717/peerj.21726/supp-5 Supplemental Information 5 Comparison between variant calling strategies

## Additional Information and Declarations

### DNA Deposition

The following information was supplied regarding the deposition of DNA sequences: The raw sequence data are available at NCBI BioProject: PRJNA680166.

### Data Availability

The following information was supplied regarding data availability: The tidyGenR data and code are available at GitHub and CRAN: - https://github.com/csmiguel/tidyGenR
- https://doi.org/10.32614/CRAN.package.tidyGenR
The data and code are available at GitHub and Zenodo: - https://github.com/csmiguel/tidyGenR_benchmarking
- https://csmiguel.github.io/tidyGenR_benchmarking/
- Camacho, M. (2026). tidyGenR-benchmarking [Dataset]. Zenodo. https://doi.org/10.5281/zenodo.18865722
The raw sequence data are available at NCBI BioProject: PRJNA680166.
