# Supplementary material for: tidyGenR: tidy multilocus amplicon genotypes in R
Source: PeerJ. 2026 Sep 17;14:e21726. doi: 10.7717/peerj.21726 (PMC13589960; doi:10.7717/peerj.21726)
Supplement: Supplementary file 1 [file peerj-14-21726-s002.pdf]

Supplementary Figures: tidyGenR: tidy multilocus amplicon genotypes in R

Miguel Camacho-Sanchez and Jennifer Leonard

2026-03-04

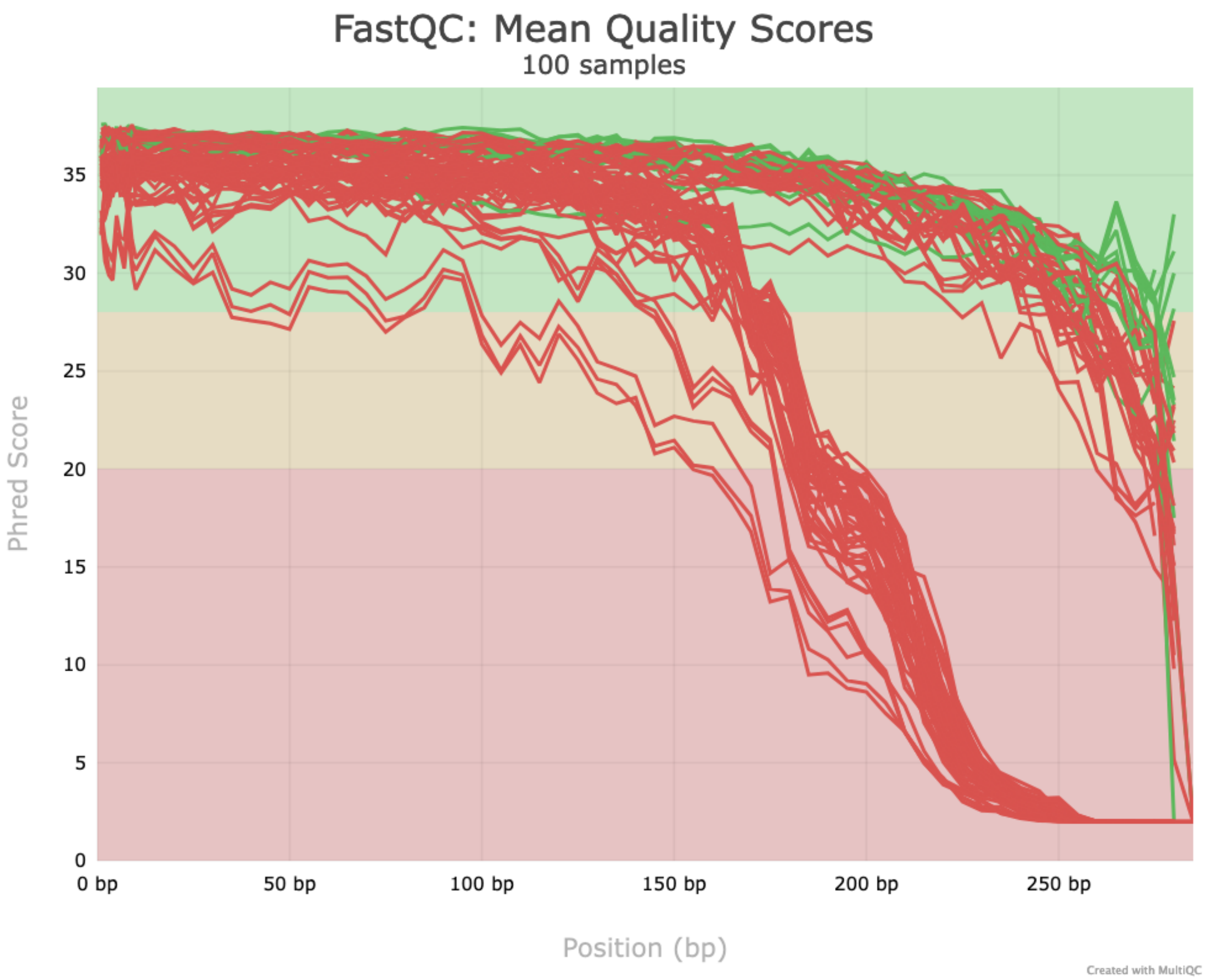

Supplementary Figure 1: Read quality of randomly selected FORWARD and REVERSE demultiplexed reads.

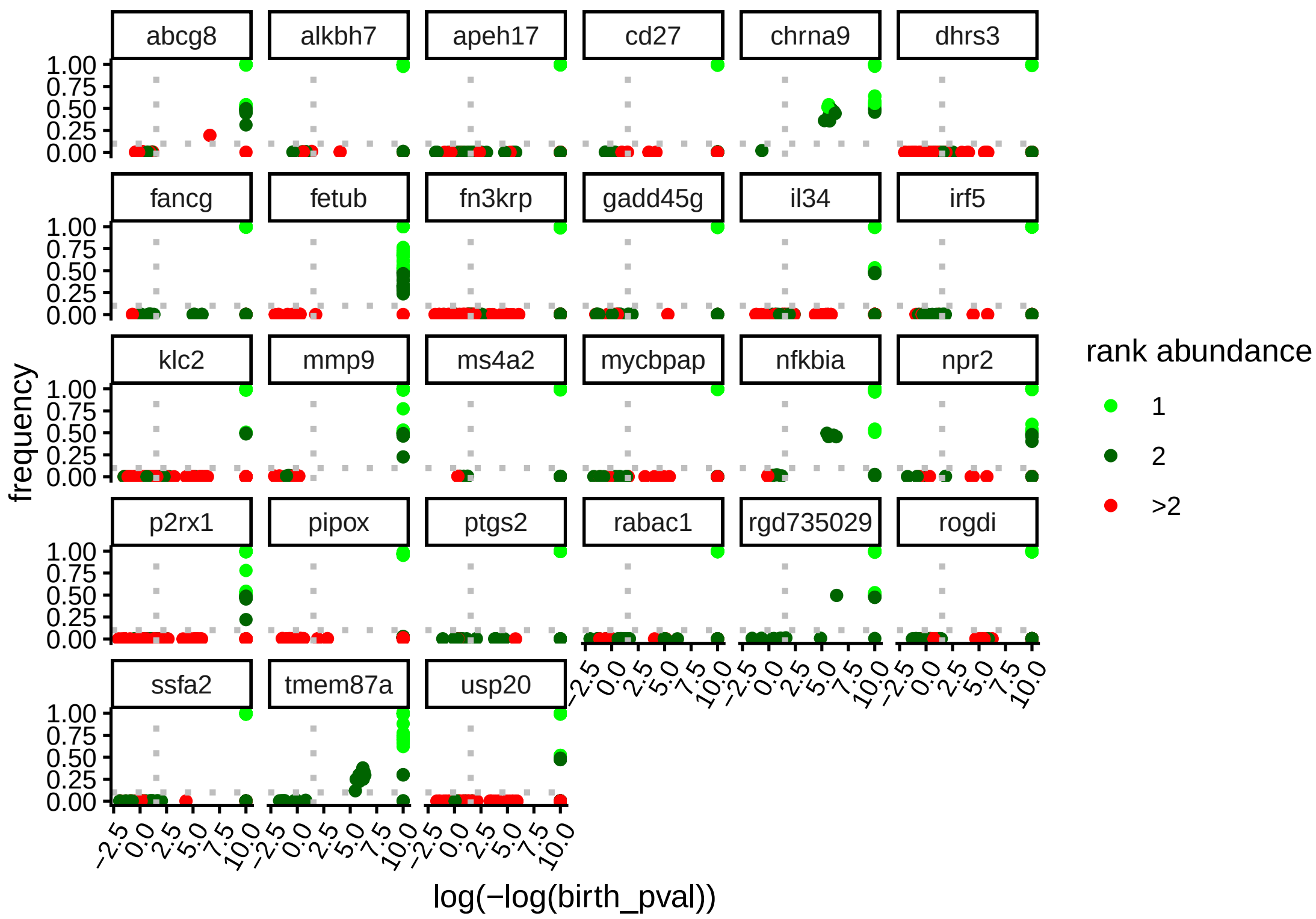

Supplementary Figure 2: Exploration of DADA2 clustering for FORWARD reads using OMEGA_A = 0.9, BAND_SIZE = 0, and pool = F. The Y-axis represents the frequency of the variant in a given locus and sample. The log(-log(birth_pval)) transformation in the X-axis is a handy way to represent the p-value of a variant being significantly overabundant. For representation purposes a birth_pval of 0 (thus negative infinite), is converted to 10. Points are color coded according to the variant rank in read abundance for its given locus and sample.

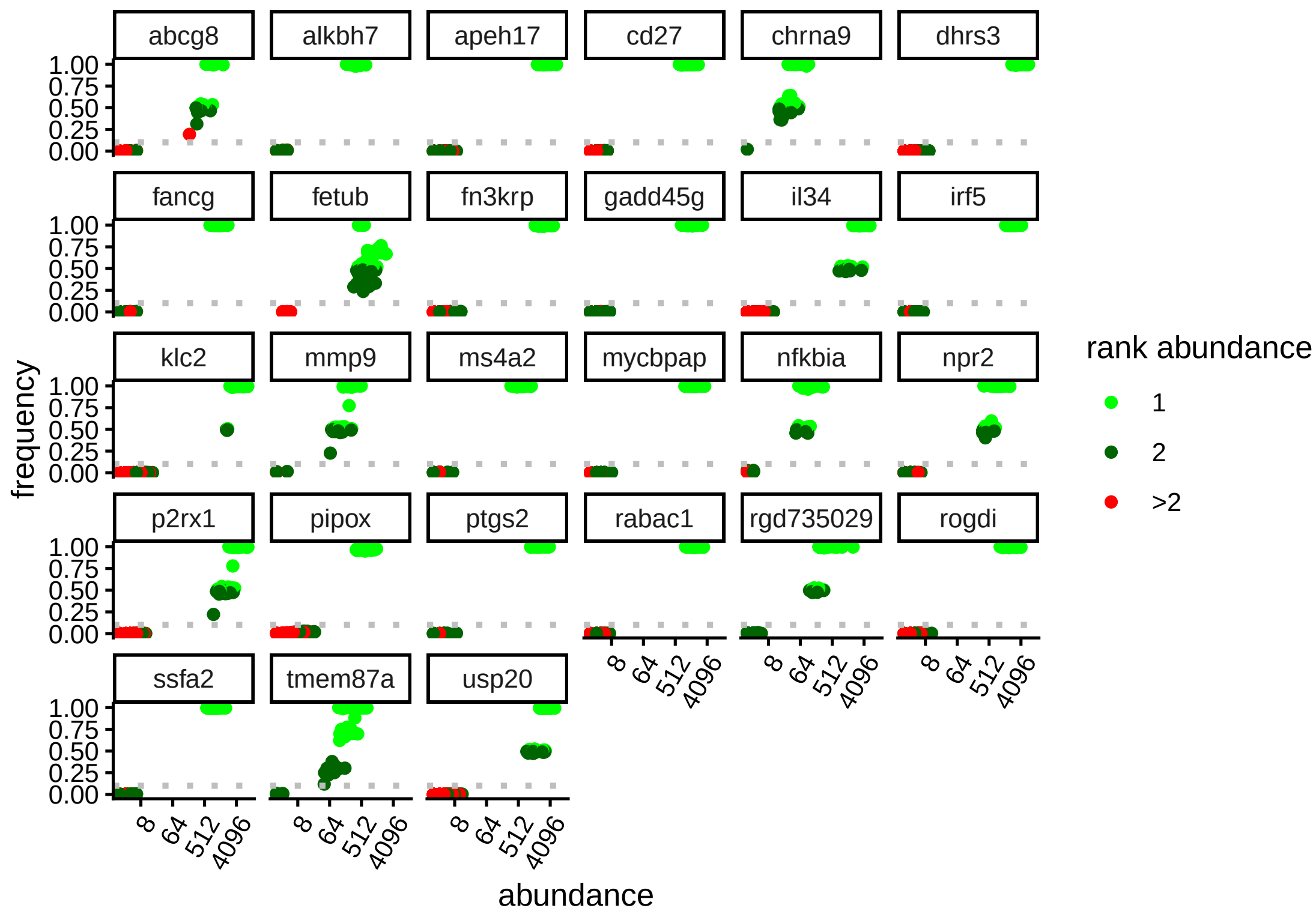

Supplementary Figure 3: Exploration of DADA2 clustering for FORWARD reads using OMEGA_A = 0.9, BAND_SIZE = 0, and pool = F. The Y-axis represents the frequency of the variant in a given locus and sample. Abundance in the X-axis is in log10 scale. Points are color coded according to the variant rank in read abundance for its given locus and sample.

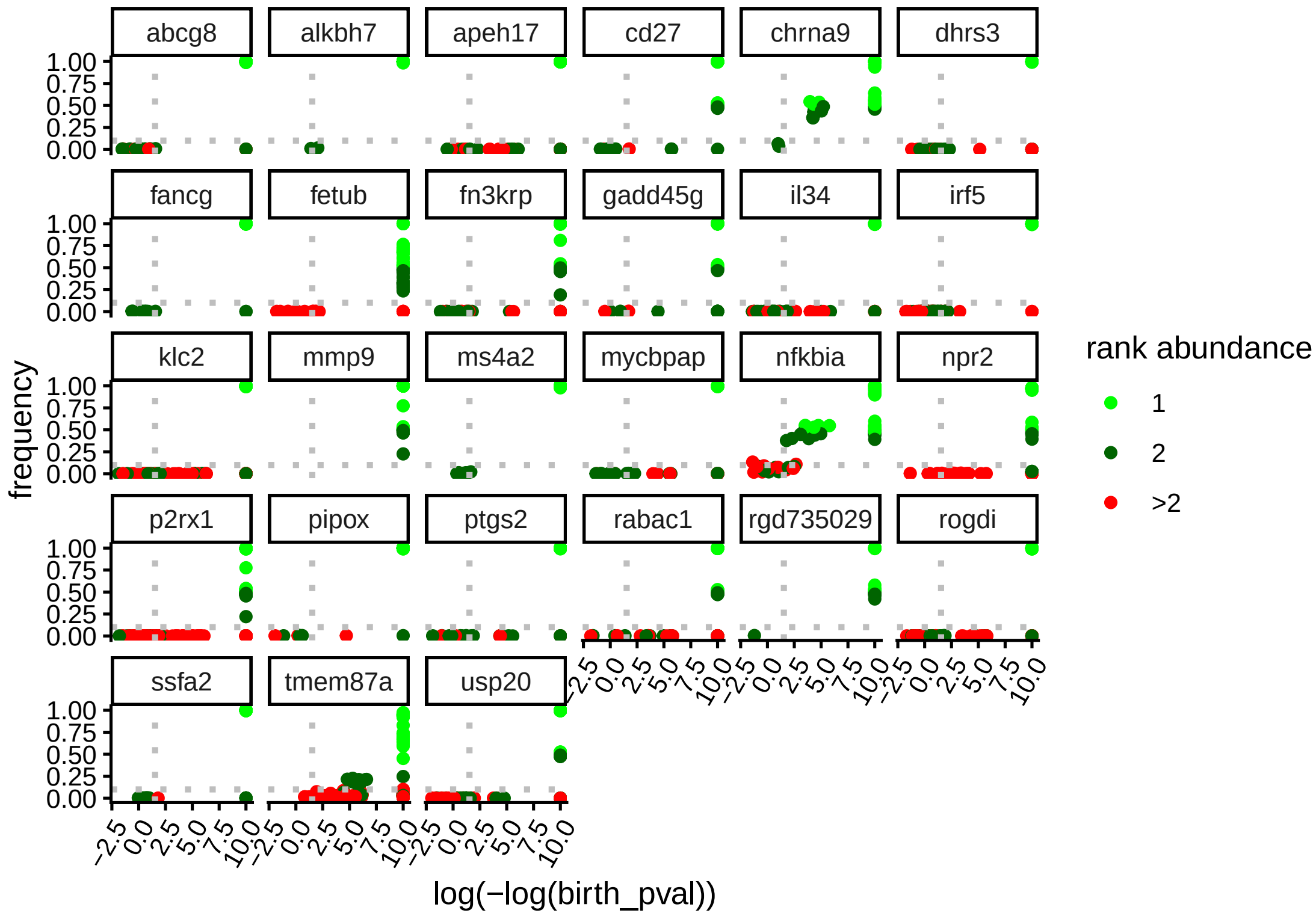

Supplementary Figure 4: Exploration of DADA2 clustering for REVERSE reads using OMEGA_A = 0.9, BAND_SIZE = 0, and pool = F. The Y-axis represents the frequency of the variant in a given locus and sample. The 'log(-log(birth_pval))' transformation in the X-axis is a handy way to represent the p-value of a variant being significantly overabundant. For representation purposes a "birth_pval" of 0 (thus negative infinite), is converted to 10. Points are color coded according to the variant rank in read abundance for its given locus and sample.

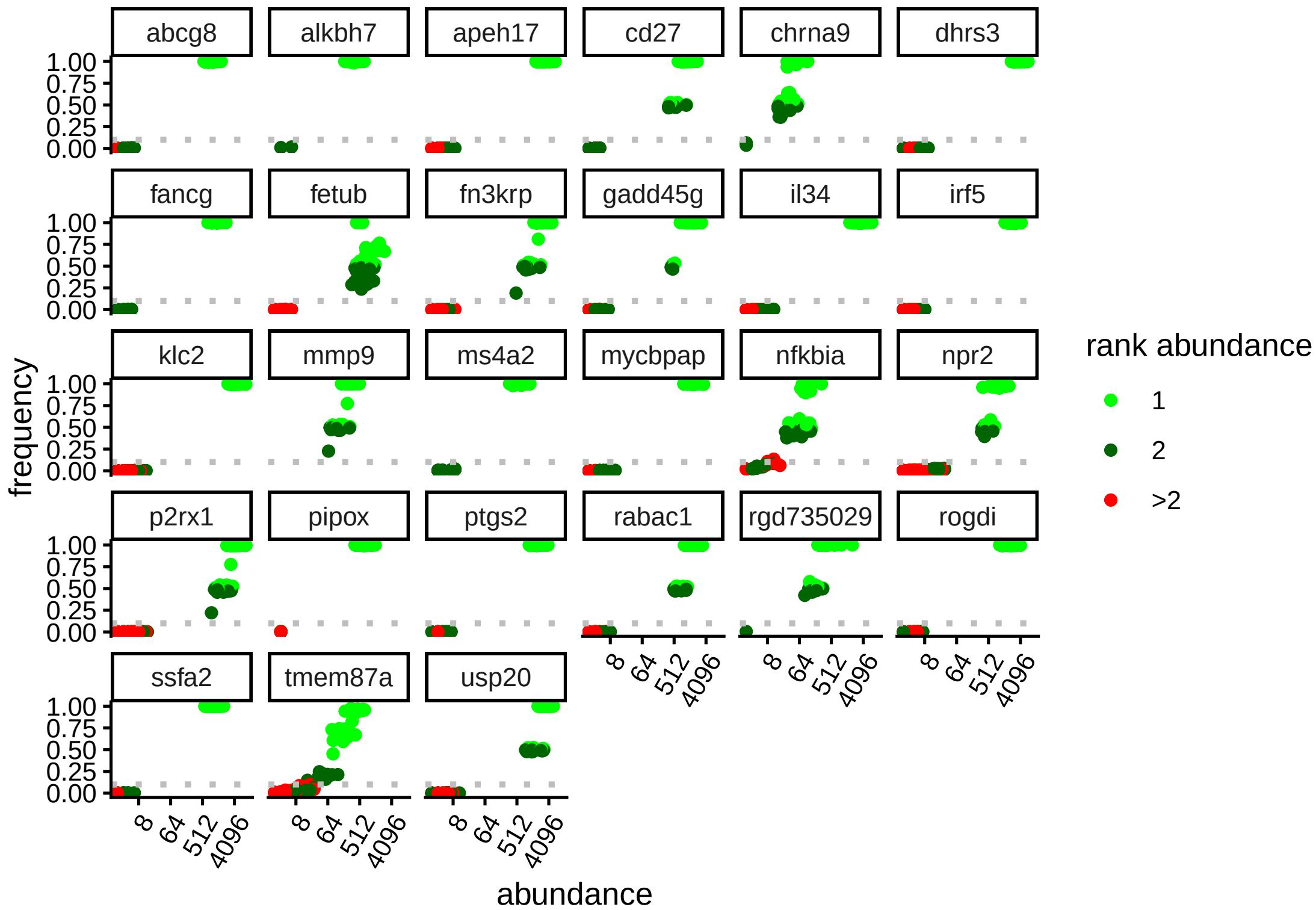

Supplementary Figure 5: Exploration of DADA2 clustering for REVERSE reads using OMEGA_A = 0.9, BAND_SIZE = 0, and pool = F. The Y-axis represents the frequency of the variant in a given locus and sample. Abundance in the X-axis is in log10 scale. Points are color coded according to the variant rank in read abundance for its given locus and sample.

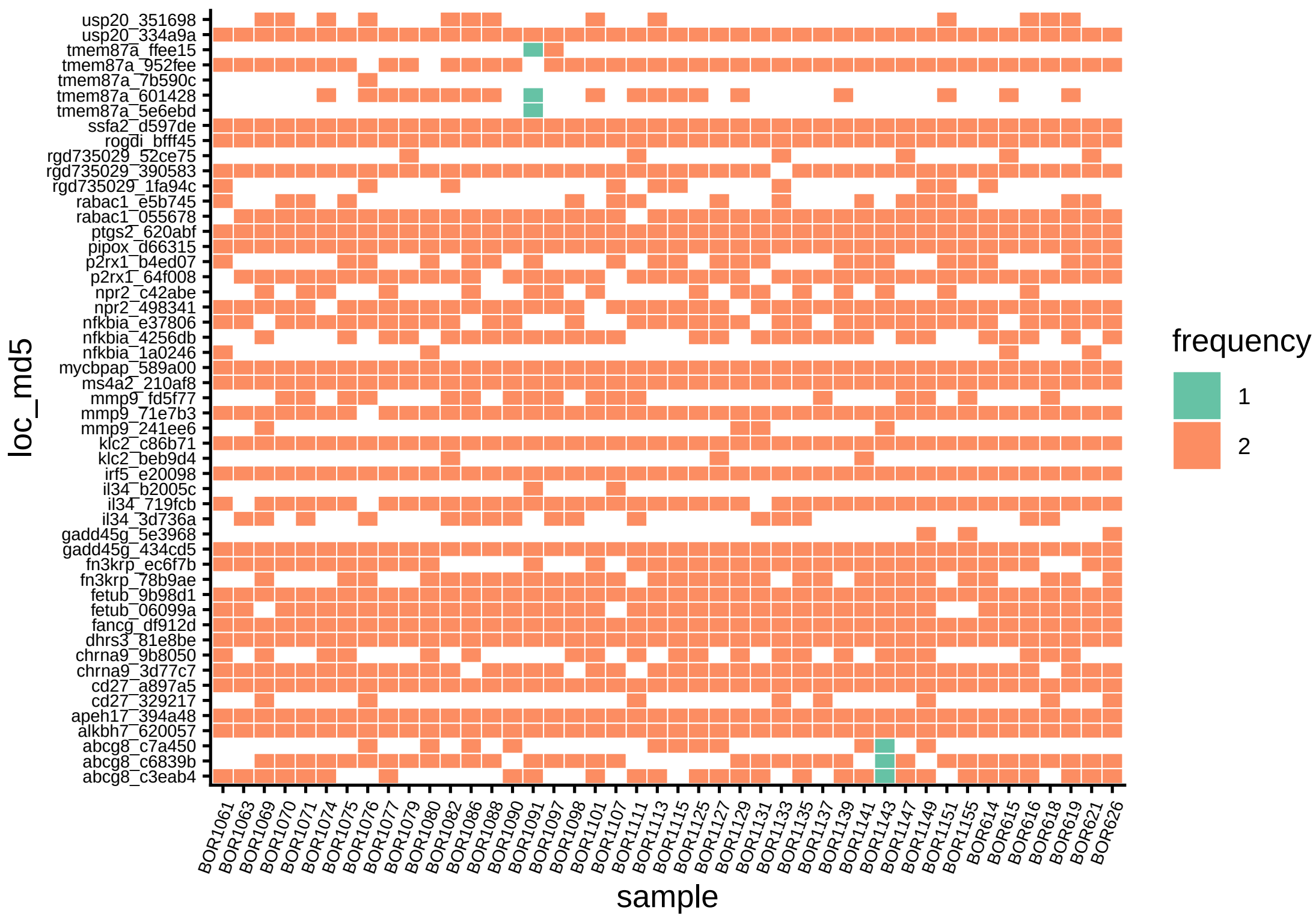

Supplementary Figure 6: Comparison of the genotypes against their respective variants after being called forcing genotypes (ADt = 10, ploidy = 2) for the selected strategy pool = F, OMEGA A = 0.01, BAND SIZE = 0. Variants are listed on the Y-axis coded as locus name followed by the first six characters of the MD5 hash of the DNA sequence. FREQUENCY = 1, indicates it was only detected as a variant but not as a genotype. The variants ared dropped due to the presence of 3 alleles, being the maximum of expected alleles = 2.

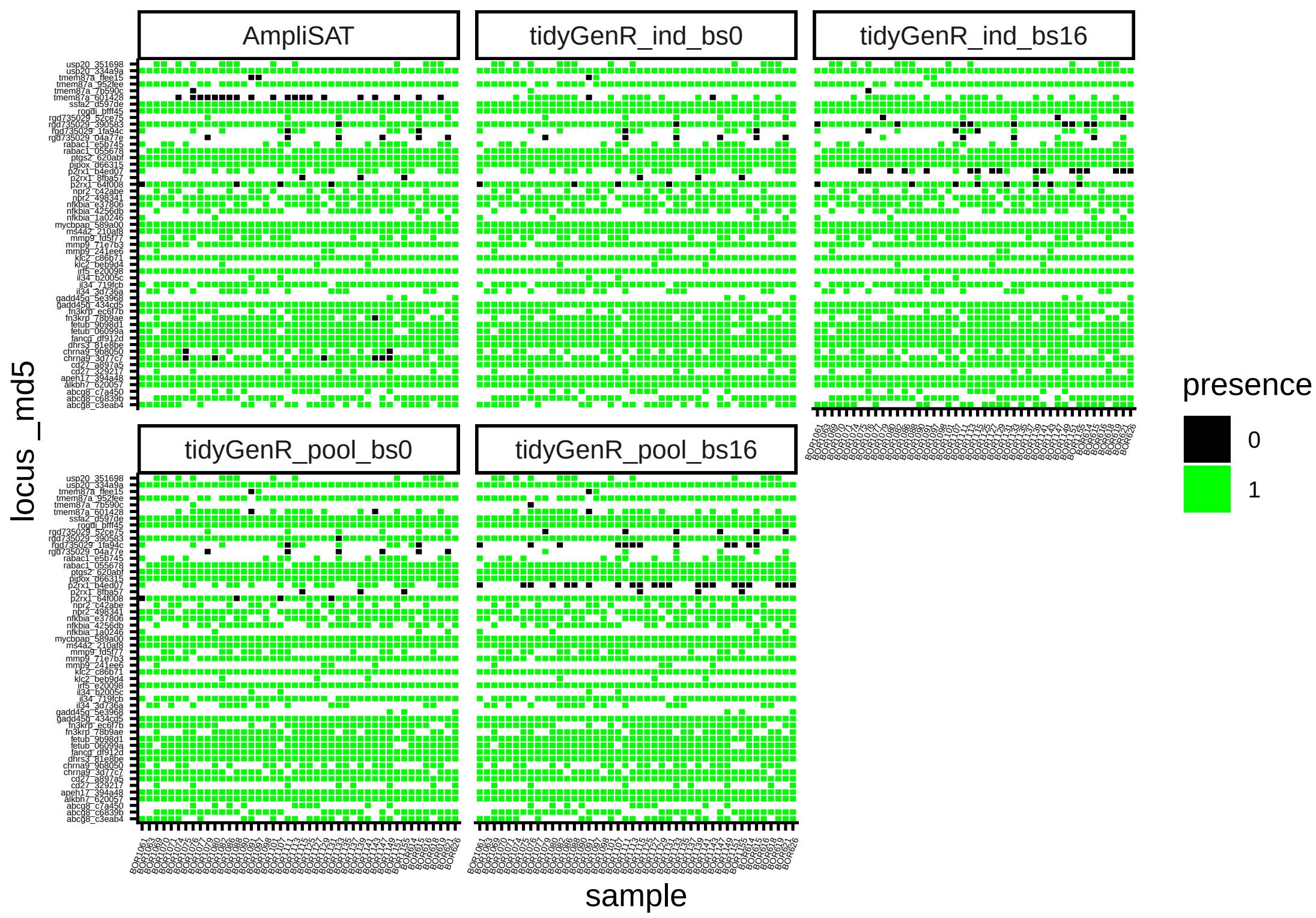

Supplementary Figure 7: Variants called with AmpliSAT (top-left), and multiple strategies with tidyGenR, combining band sizes of 0 (bs0) and 16 (bs16), individual (ind) and pooled (pool) calls. OMEGA_A was set to 0.01 in all cases with tidyGenR. Green tiles are identified alleles. Black tiles represent alleles not present in the given dataset but present in any of the other cases compared. Alleles are listed on the Y-axis coded as locus name followed by the first six characters of the MD5 hash of the DNA sequence.

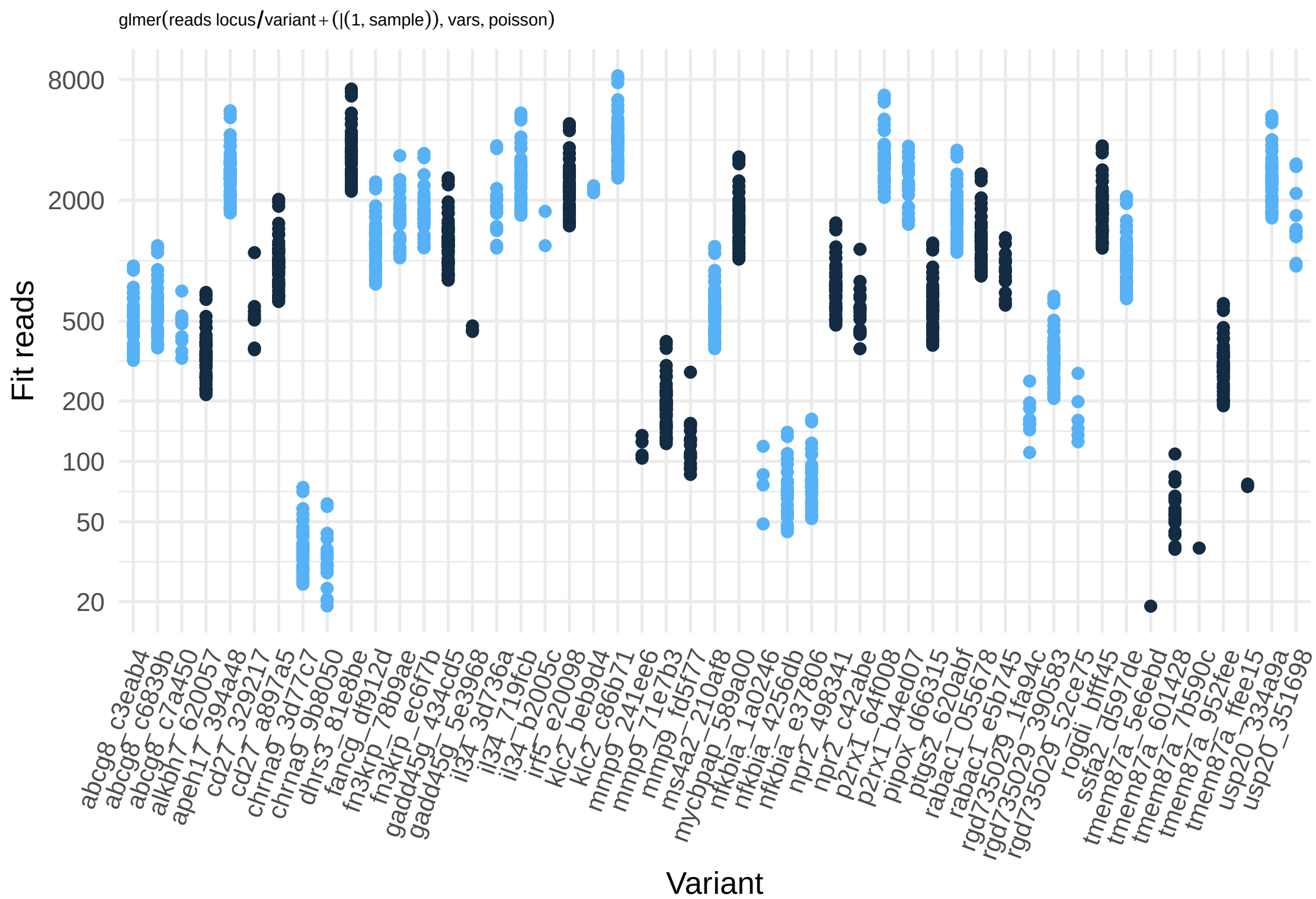

Supplementary Figure 8: Predicted values for reads in each variant from a fitted mixed model: glmer(reads locus/variant + (1 | sample), data = variants, family = "poisson"). Variants are named with locus name followed by the six first characters of the MD5 hash of its corresponding DNA sequence.

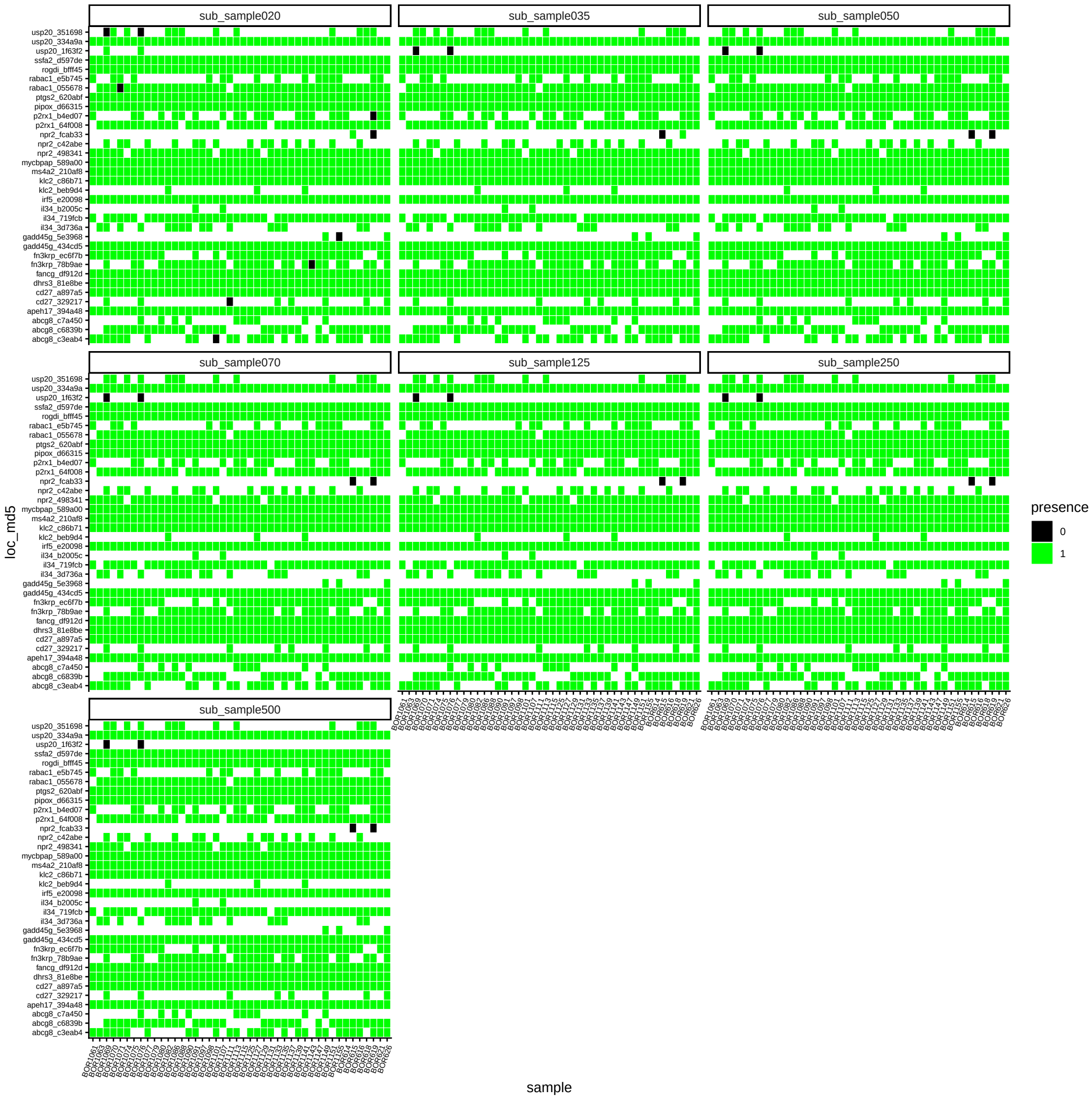

Supplementary Figure 9: Effect of coverage across loci/samples. Genotypes called over 20 loci on donwsampled FASTQ files at 500x, 250x, 125x, 70x, 50x, 35x, and 20x.Green tiles are identified alleles. Black tiles represent alleles not present in the given dataset but present in any of the other cases compared. Alleles are listed on the Y-axis coded as locus name followed by the first six characters of the MD5 hash of the DNA sequence.

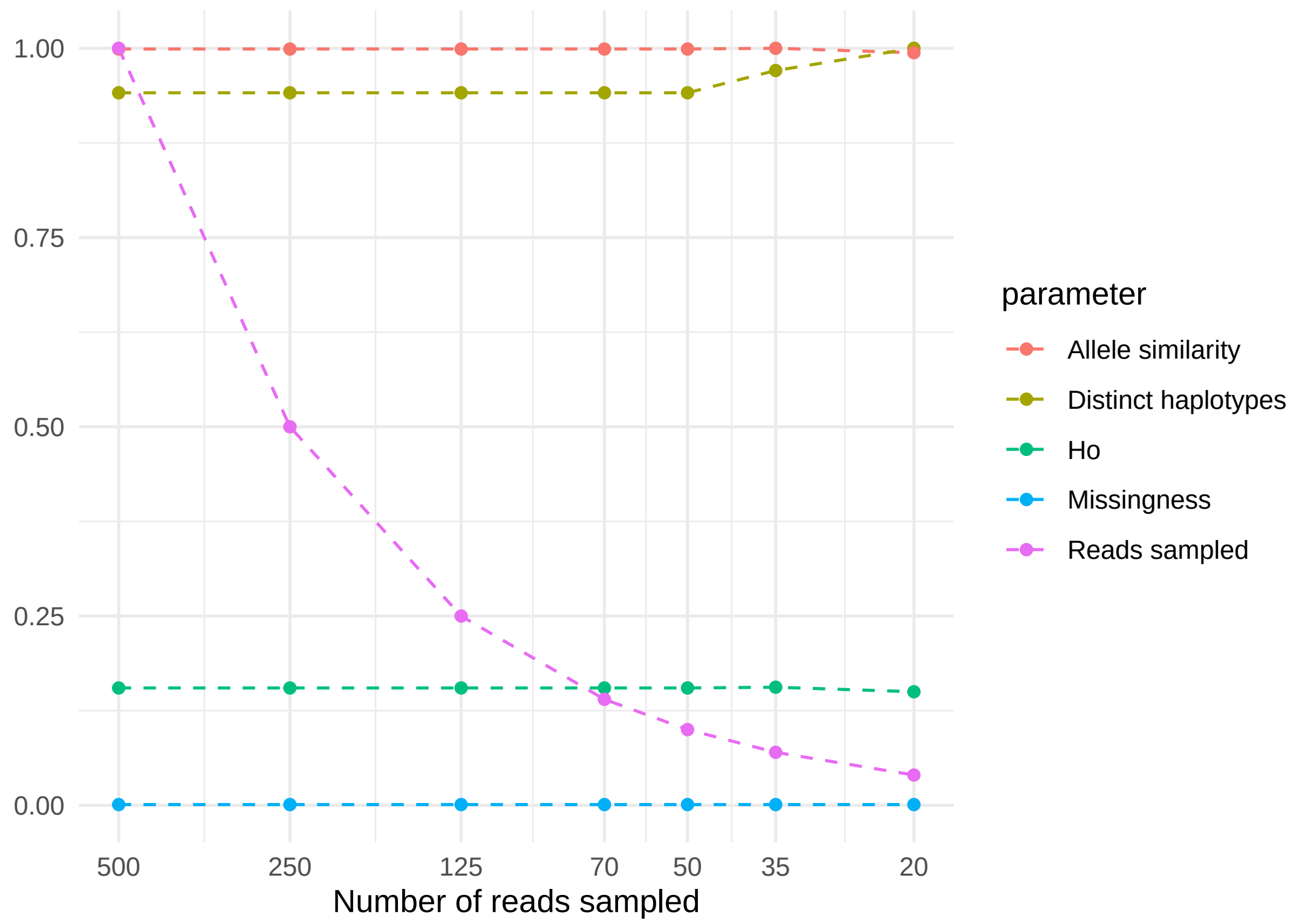

Supplementary Figure 10: Effect of coverage summarised by statitics on number of haplotypes, missingness, heterozygosity, and similarity of allele calls (definitions in the main text).

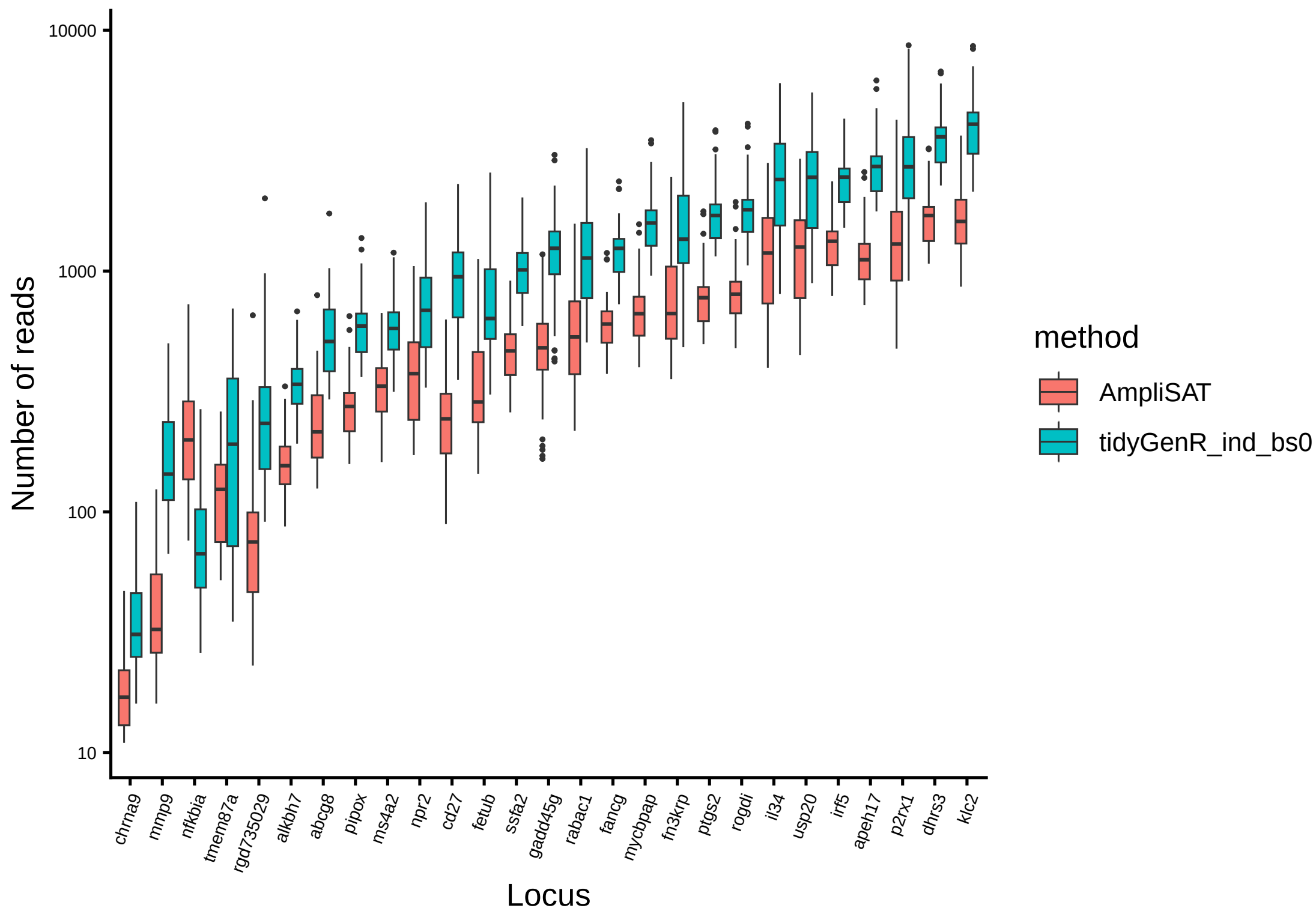

Supplementary Figure 11: Comparison of the number of reads supporting filtered variants in each locus for AmpliSAT ('amplisas') and tidyGenR ('ind_bs0').
